# Neurology

**Published:** 1900-10

**Authors:** William C. Krauss

**Affiliations:** Buffalo, N. Y.


### NEUROLOGY.

CONDUCTED BY WILLIAM C. KRAUSS, M. D., Buffalo, N. Y.

AT THE thirteenth International Medical Congress, held at Paris, August 2-9, 1900, the section on neurology presented a very excellent program. Some of the papers read are abstracted as follo.vs:

### THE NON-TABETIC LESIONS OF THE POSTERIOR COLUMNS.

By M. BRUCE, M. D., Edinburgh, Eng.

The subject was discussed in two sections: I. Anatomical. II. Pathological.

I. The constitution of the posterior columns, as shown by degenerations following: (1) lesions of nerve roots; (2) lesions of the cord itself, interrupting the continuity of the posterior columns; (3) lesions of the gray matter.

*Exogenous Tracts.*— Differentiation of Goll's and Burdach's columns depends on termination of their long fibers in the nucleus gracilis and nucleus cuneatus respectively. Burdach's column commences at fifth dorsal segment. Goll's column as a definite and characteristic form at each segment, any departure from which indicates a partial involvement of its components. Kahler's law regarding the positions of the exogenous fibers in the columns are generally true, but requires some modification for the cervical and dorsal regions. It is applicable to descending branches of the roots also. The relative position of ascending fibers of roots, as far as known, will be considered. Descending exogenous fibers in the cervico-dorsal region occupy the position of the comma tract of Schultze in part of their course. The descending root fibers from lower dorsal, lumbar and sacral roots enter the septo-marginal tract in part.

*Endogenous Tracts.*—Cornu-commissural zone of Marie, comma tract of Schultze, septo-marginal tract (including the oval field of Flechsig and the triangular area of Gombault and Philippe), the descending tract of Hoche, and the postero-internal root-zone of Flechsig will be considered. It will be shown that they are not purely endogenous but contain exogenous fibers also. The endogenous fibers of the cornu-commissural zone are short and degenerate upwards. Those of the comma tract degenerate downwards for several segments. Those of the septo-marginal tract are derived from segments as high as the eighth cervical and degenerate downwards. The postero-internal root-zone is mainly composed of root fibers of indeterminate length which enter it horizontally in the sacral and dorsal regions and in the lumbar and cervical regions pass obliquely upwards into it.

The areas of Flechsig will be considered.

II. The pathological changes in the posterior columns considerd in relation to the diseases which involve them.

*a.* Progressive muscular atrophy of the Charcot—Marie type. Sclerosis in posterior columns throughout the cord (with the exception of the sacral region); involving the columns of Burdach, and to a lesser extent the columns of Goll; leaving a healthy zone along the posterior commissure and posterior cornu, and partially also in the postero-internal root-zone. Lissauer's tract generally involved in the sclerosis. Posterior roots degenerated.

*b.* Cerebral tumors frequently produce degenerations in the posterior columns, sometimes interpreted as due to retrograde degeneration from the nuclei of the columns. These are probably due to ascending degenerations of one or more roots which have been injured at the point of their entrance in the cord, either through tension produced in the leptomeninges by accumulation of cerebro-spinal fluid or by the action of toxins on the vulnerable point of Redlich and Obersteiner.

*c.* Syringomyelia.—Affected areas are: (1) behind the posterior commissure, (2) along the posterior median septum, (3) a narrow strip between Goll and Burdach's columns. The lesions are probably due to a direct extension from the gray matter, and not to secondary degeneration from involvement of trophic centers within the gray matter. Destruction of these areas, however, may produce secondary degeneration.

*d.* Friedreich's ataxia.—The lesion is a sclerosis in the posterior and antero-lateral columns. So far as the posterior columns are concerned, this sclerosis is most marked in the lower dorsal regions and diminishes upwards and downwards; being practically absent in the sacral region, and ceasing entirely at the nuclei of the posterior columns. The sclerosis presents an exuberant growth of neuroglia usually arranged in whorls and in interlacing fasciculi. It involves the greater part of the column with the partial exception of the cornu commissural and the septo-marginal tracts. The pia-mater is usually normal and the vessels show slight or no thickening. The posterior roots show a similar change with various stages of attenuation and demyelinisation of their fibers. Change in the sensory peripherical nerves and atrophy of the posterior root ganglia suggest that the nature of the process is a primarily imperfect development of the sensory neuron with subsequent degeneration, and secondary increase of neuroglia.

*e.* Combined sclerosis of the posterior and antero-lateral columns —The term includes several groups of diseases which agree in the simultaneous degenerations and sclerosis of these columns (these changes being not, however, accurately limited to definite systems), but which differ from each other in their clinical course and in their etiology. The most important diseases are pellagra, ataxic paraplegia (Gowers) or ataxo-paraplegic tabes (Déjèrine) combined system degeneration of German authors, the combined degeneration in grave anemia, subacute combined degeneration recently described in England, etc. It is certain that all these titles do not indicate distinct morbid entities.

In pellagra, the sclerosis, according to Tuczek, affects chiefly the column of Goll and the middle root-zone, and spares relatively the cornu-commissural and the septo-marginal tracts. The roots, according to Tuczek, are healthy; according to Babes, they are degenerated. Both these observers regard the sclerosis as the result of the destruction of the nerve fibers by the direct action of some toxins. It has also been regarded as secondary to a degeneration of the endogenous fibers, due to vascular changes in the gray matter.

In ataxic paraplegia the lesions resemble those of subacute combined degeneration, but the disease differs from the latter in its much more prolonged course.

The degeneration of the columns in grave anemia seem to be the same as those described by Rothmann as combined system-disease and by Russel, Collier and Batten, as subacute combined degeneration. The lesion is most marked in dorsal and cervical region. It affects mainly the middle root-zone in the lumbar region, and in the cervical and dorsal, chiefly Goll's columns. The area near the posterior commissure and the posterior cornu, and, in the lumbar region, that next the septum, are spared. The fact that the degeneration spreads round the periphery of the cord irrespective of systems and that the posterior roots are spared and the membranes healthy, indicate a vascular origin to the process which is probably of toxic nature. In anemic cases the cord change is probably not the result of the anemia but due to its cause.

---

### THE DIFFERENTIAL DIAGNOSIS OF ORGANIC AND HYSTERICAL HEMIPLEGIA.

By DAVID FERRIER, M. D., London, Eng.

Hysterical hemiplegia may simulate hemiplegia due to organic cerebral disease, and there is scarcely a single symptom uniformly present sufficient of itself to enable one, off hand, to differentiate the one from the other.

A correct diagnosis is, however, in most cases possible, and this depends on a consideration of several factors, of which the more important are the following:

(1) The personal and family history; (2) the mode of onset; (3) the characters of the paralysis; (4) the course and termination; (5) the state of the superficial and deep reflexes.

*a.* The personal and family history.

Hysterical hemiplegia, like organic hemiplegia, may occur at all ages. The subjects of hysterical hemiplegia are individuals, male and female, of neurotic inheritance, and of the so-called hysterical temperament, and who have either been previously liable to hysterical attacks, or exhibit some of the permanent stigmata of hysteria, such as zones of hyperesthesia, or anesthesia or complete sensitivo-sensorial hemianesthesia of the recognised type (Charcot.)

The subjects of organic hemiplegia are (apart from traumatism, or autochthonous intracranial lesions) those predisposed by vascular, renal, or cardiac disease to cerebral hemorrhage, embolism or thrombosis, of the existence of which it is generally possible to obtain evidence by careful clinical investigation.

*b.* The mode of onset.

Hysterical hemiplegia occurs most commonly after some great nervous disturbance, such as emotional shock, or after an epileptiform or apoplectiform attack (so-called hysterical apoplexy) simulating cerebral hemorrhage. But hysterical apoplexy is probably only a phase of the grand hysterical attack, and differs from true apoplexy *inter alia* in the absence of disturbance of the circulation, respiration and temperature.

*c.* The characters of the paralysis.

In hysterical hemiplegia, the upper and lower extremity may be more or less completely powerless and flaccid, but the face is rarely so. Such affection of the face as occurs, is usually of the labio-glossal spasmodic type (Charcot) on the same or opposite side. The leg is usually more affected than the arm, and in walking the paralysed leg is dragged like an inert mass, and not circumducted as in organic hemiplegia (Todd's symptom).

In the majority of cases hysterical hemiplegia is associated with complete sensitivo-sensorial hemianesthesia. In organic hemiplegia, accompanied by hemianesthesia from lesion involving the sensory tracts of the internal capsule, the loss of sensation is rarely profound, the special senses of hearing, smell and taste are rarely impaired together, and when vision is affected it is usually in the form of hemianopsy, and not of crossed amblyopia with concentric contraction of the visual fields as in hysteria.

Hysterical monoplegia not infrequently follows local traumatism after a longer or shorter interval (Charcot), and differs from cortical monoplegia in its absolute restriction to one limb, or one segment of this limb, and in the association of the motor paralysis with anesthesia, which is morphological in character (Charcot) and does not correspond to the peripheral distribution of any sensory nerve, or posterior root of a spinal segment.

*d.* Course and termination.

Hysterical hemiplegia (monoplegia) may last an indefinite time, and continue of the same flaccid character throughout. In organic hemiplegia of more than three months duration, late rigidity or contracture occurs in the paralysed limbs, and gives place only to muscular atrophy. This contracture sets in gradually, and never develops suddenly as in hysteria.

Hysterical hemiplegia is liable to great variations, and may at any time suddenly cease.

*e* The state of the superficial and deep reflexes.

In hysterical hemiplegia the deep reflexes are not necessarily altered, and true ankle clonus is rare; whereas in organic hemiplegia the deep reflexes become unavoidably exaggerated, and ankle clonus is the rule.

In hysterical hemiplegia, the plantar reflex is usually absent or difficult to obtain. If it can be elicited, it is of the normal "flexor" type. In organic hemiplegia (and in all lesions of the pyramidal tracts) the plantar reflex is of the "extensor" type (Babinski's symptom: phénomène des orteils).

### NATURE OF TENDONS-REFLEXES.

By C. H. SHERRINGTON, M. D., Liverpool, Eng.

Two distinct sets of phenomena are included under this name :

1. True spinal and spino-cerebral reflexes from tendons.

2. Pseudo-reflexes commonly known as tendon phenomena or by British and American writers as "jerks."

The former, *i. e.*, No. 1, are easy of explanation. The tendons of muscles, long ago recognised by Bichat as capable of affecting sensation, contain the end-organs of afferent nerves. These are the end organs described by Golgi, by Ruffini and others. These organs can be excited by mechanical means, probably mechanical strain is the stimulus which is their normal and adequate mode of excitation.

The true tendon reflexes are not so much importance to clinical medicine as are the pseudo-reflexes (tendon-phenomena, "jerks.")

The latter, *i. e.*, No. 2, are typified by the "knee-jerk." An objection to calling them "tendon-phenomena" is that the tendon is not essential to the phenomenon. That they are not true reflex is shown by the time of the latency of the reaction being so brief as to exclude the possibility of reaction through a nerve-center. The "jerk" is a direct response by the muscle to sudden mechanical strain. It is only when the excitability of the muscle is great that this direct response can be elicited from it. When the muscle is cut off from the spinal motor neurons which innervate it, its excitability falls too low for the response to be possible. When the afferent spinal roots connected with the spinal tonus of the muscle are severed the excitability of the muscles also falls too low for the direct response to sudden mechanical strain. There is therefore necessary for the "jerk" the maintenance of the spinal tonus of the muscle. The reflex arc on which the spinal tonus of the muscle depends is composed of the afferent nerve-fibers coming from the muscle (*vasto_ crureus*, in the fall of the "knee-jerk") itself, and of the motor neurons innervating that muscle. The activity of that reflex arc can be exalted or inhibited by the activity of various other spinal and spino-cerebral arcs. Removal of the cerebral hemispheres leads immediately to very great exaltation of the tonus of the *vasto-crureus* muscle traceable to exalted activity of spinal motor neurons innervating that muscle. The knee-jerk is then much exalted so that a rhythmic series of jerks may follow a single tap on the patellar tendon.

On the other hand, the activity of spinal motor neurons innervating the *vasto-crureus* may be depressed by exciting activity of the motor neurons which innervate the antagonistic muscles, the flexor muscles of the knee. The activity of these motor neurons of the flexors of the knee is habitually associated with some degree of inhibition of the extensor muscles of the knee. The most easy spinal reflex to obtain in the hind limbs by excitation of the limb itself is flexion of the limb at knee and at hip. Hence an easy way of causing inhibition of the knee jerk is to excite a reflex movement of the hind limb from some portion of the limb because the flexors of the knee are brought into play and the activity of the motor cell of the extensors is then partly or completely inhibited. The inhibition can be peculiarly obtained by stimulating the flexor muscles themselves, *e. g.*, semi-membranosus.

---

### THE NON-TABETIC LESIONS OF THE POSTERIOR COLUMNS OF THE SPINAL CORD.

By CHARLES L. DANA, M. D., New York.

The posterior columns of the spinal cord and the posterior spinal roots, so far as diseases may be concerned, are studied from three points of view:

1. Diseases in connection with the embryological areas.
2. Diseases in connection with the exogenous and endogenous fibers.
3. Diseases connected the vascular supply.

The writer gives a brief review of the non-tabetic lesions. He then discusses the subject of acute ataxia due to spinal lesions, *acute spinal ataxia*. He gives the observations of Leyden and others as to acute ataxia due to bulbar lesions and describes a distinct class of cases due to acute lesions of the posterior columns of the cord. These occur in elderly or aged people with generally a specific history. Personal cases are reported and literature referred to. He gives his views as to the pathology of the disease.

The writer next discusses the subject of lesions of the posterior columns due to cachectic, anemic and infectious states. He describes the condition which he calls "subacute spinal ataxia," due to lesions of the posterior, and often of the lateral columns. The etiology, course and pathology of this disorder is described from the personal experience of the writer, based upon a study of sixteen cases of his own, in three of which there were autopsies.

